# Bibliometric analysis of the top 100 highly cited articles on sublobectomy for non-small cell lung cancer

**DOI:** 10.1186/s13019-024-02854-0

**Published:** 2024-06-26

**Authors:** Chao Guo, Lei Liu, Jiaqi Zhang, Ke Zhao, Yeye Chen, Cheng Huang, Shanqing Li

**Affiliations:** Department of Thoracic Surgery, Peking Union Medical College Hospital, Shuaifuyuan 1, Wangfujing Street, Dongcheng District, Beijing, P. R. China

**Keywords:** Non-small cell lung cancer, Sublobectomy, Bibliometric analysis, Citations, Recurrence, Clinical trial

## Abstract

**Objectives:**

The goal of this research is to pinpoint the top 100 most frequently referenced studies on sublobectomy for non-small cell lung cancer.

**Methods:**

We identified the top 100 most frequently referenced studies on sublobectomy for non-small cell lung cancer by searching the Web of Science database. We extracted key information from the selected studies, including the author, journal, impact factor, type of article, year of publication, country, organization, and keyword.

**Results:**

To the best of our understanding, this is the inaugural bibliometric study on sublobectomy for non-small cell lung cancer. The publication years of the top 100 most frequently referenced studies span from 1994 to 2022, with citation counts ranging from 51 to 795. The majority of the included studies are original (93/100) and primarily retrospective studies (82/93). The United States leads in terms of published articles and citations, with the Annals of Thoracic Surgery being the most frequently sourced journal (*n* = 27). High-density keywords primarily originate from limited resection, lobectomy, survival, carcinoma, recurrence, randomized trial, radiotherapy, lung cancer, outcome, 2 cm, as revealed by CiteSpace analysis.

**Conclusions:**

Our research compiles and analyzes the top 100 most frequently referenced studies in the field of sublobectomy for non-small cell lung cancer. The United States has the most published and cited works on this topic. Currently, the hot keywords for sublobectomy research are gradually shifting towards prognosis and obtaining better evidence-based medical evidence to demonstrate its value in the treatment of non-small cell lung cancer.

## Introduction

Lung cancer is one of the leading causes of cancer-related deaths worldwide, accounting for approximately 1.8 million new cases and 1.6 million deaths each year [1]. Surgical resection remains the primary treatment for early-stage non-small cell lung cancer (NSCLC), with lobectomy being the standard procedure for most patients [2]. However, for patients with compromised lung function or other comorbidities, a less invasive surgical approach, such as segmentectomy or wedge resection, may be more appropriate [3]. Segmentectomy, also known as sublobectomy or anatomical segmental resection, involves the removal of a specific bronchopulmonary segment, preserving more lung parenchyma than lobectomy [4].

In recent years, there has been a growing interest in sublobectomy as a potential alternative to lobectomy for early-stage NSCLC, particularly for patients with small, peripheral tumors [5]. Several studies have reported comparable oncological outcomes between segmentectomy and lobectomy for tumors less than 2 cm in size [6–10]. Moreover, sublobectomy has been associated with better preservation of lung function, shorter hospital stays, and fewer postoperative complications compared to lobectomy [8, 11].

Given the increasing importance of sublobectomy in the management of early-stage NSCLC, it is crucial to understand the current state of research in this field. Bibliometric analysis is a useful tool for evaluating the scientific impact of research articles and identifying trends in a specific research area [12]. To the best of our knowledge, no bibliometric analysis has been conducted on the top-cited articles related to sublobectomy in NSCLC surgery.

In this study, we aimed to perform a bibliometric analysis of the 100 most-cited articles on sublobectomy for NSCLC. We sought to identify the most influential articles, authors, institutions, and countries in this field, as well as to analyze the trends in research topics and methodologies. This information may help guide future research efforts and inform clinical decision-making in the management of early-stage NSCLC.

## Materials and methods

### Study design

This study aimed to conduct a bibliometric analysis of the top 100 highly cited articles on sublobectomy for NSCLC. In this study, “sublobectomy” is defined to include both anatomical segmentectomy and wedge resection. The bibliometric analysis was performed to identify the most influential articles, authors, institutions, and countries in this field, as well as to analyze the trends and characteristics of these articles.

### Data source and search strategy

A comprehensive literature search was conducted using the Web of Science (WoS) database to identify the top 100 highly cited articles on sublobectomy for NSCLC. The search was performed on March 12, 2023, to ensure the most recent and accurate data. The search terms used were "segmentectomy NSCLC" OR "segmentectomy non-small cell lung cancer" OR "sublobar resection NSCLC" OR "sublobar resection non-small cell lung cancer" OR "limited resection NSCLC" OR "limited resection non-small cell lung cancer" OR "sublobectomy NSCLC" OR "sublobectomy non-small cell lung cancer". The search was limited to articles published in English.

### Selection criteria

The search results were sorted by the number of citations, and the top 100 highly cited articles were selected for further analysis. The inclusion criteria were: (1) articles focusing on sublobectomy for NSCLC, (2) articles published in English. The exclusion criteria were: (1) articles not related to sublobectomy for NSCLC, (2) articles focusing on other surgical procedures or treatments for lung cancer, (3) articles published in languages other than English.

### Article selection and data extraction

The initial search results were screened by two independent reviewers based on the title and abstract. Any disagreements between the reviewers were resolved through discussion or consultation with a third reviewer. The full texts of the potentially eligible articles were then assessed for eligibility based on the inclusion and exclusion criteria. The top 100 highly cited articles were selected based on the total citation count.

The following data were extracted from the selected articles: (1) title, (2) author(s), (3) year of publication, (4) journal, (5) country of origin, (6) institution, (7) total citation count, (8) keywords, and (9) centrality of keywords.

### Data analysis

Descriptive statistics were used to analyze the data. The frequency of articles by year of publication, journal, author, institution, and country were calculated. The total number of citations were also calculated for each article.

### Network analysis

A network analysis was performed to visualize the relationships among keywords, authors, institutions, and countries of the top 100 highly cited articles. The network analysis was conducted using CiteSpace software (version 6.1.R1). The nodes in the network represented the keywords, authors, institutions, or countries, and the links between the nodes represented the co-citation or co-authorship relationships. The size of the nodes and the thickness of the links were proportional to the number of citations or co-citation/co-authorship relationships, respectively.

## Results

**Fig. 1:**
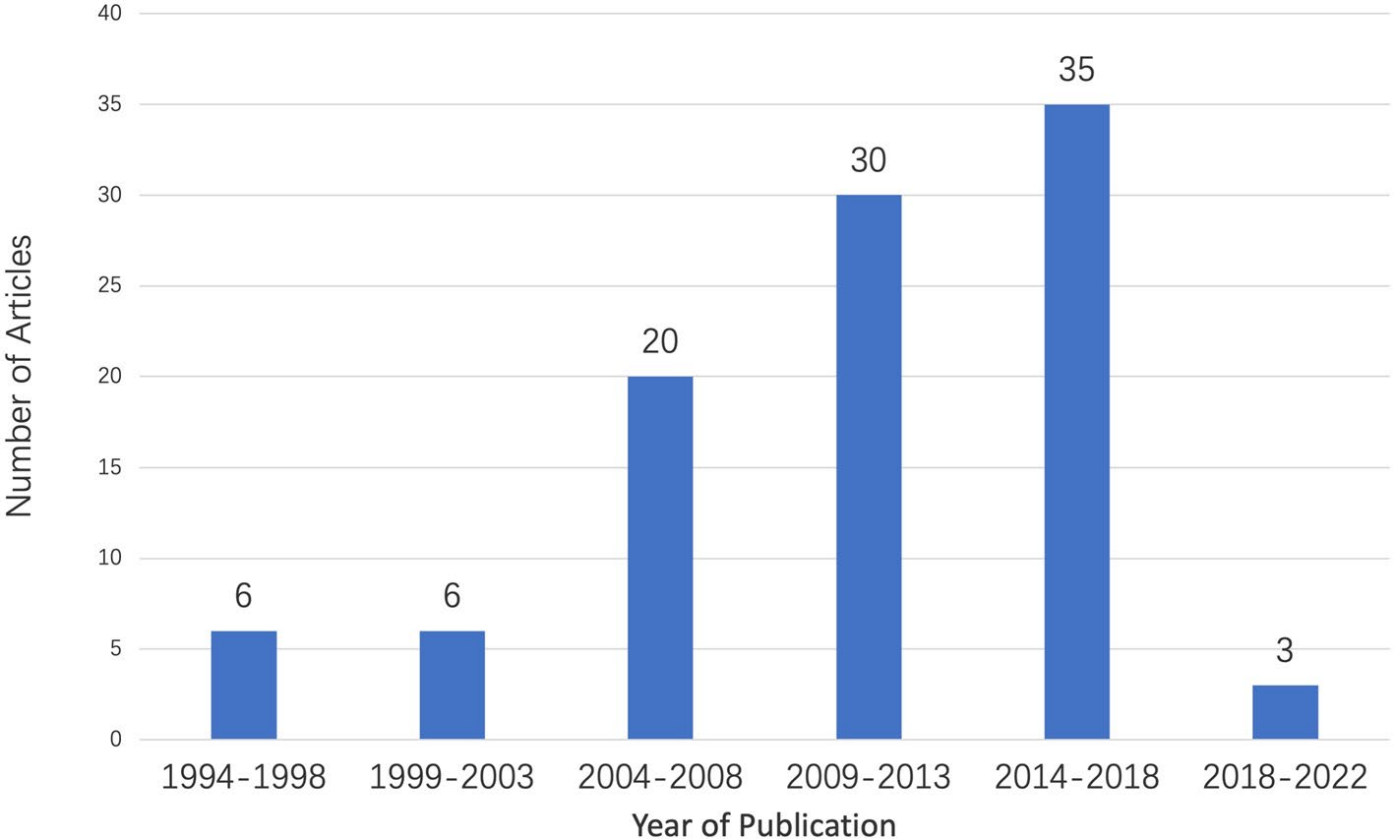
5-year interval for 100 most cited articles of sublobectomy

The top 100 most cited articles for sublobectomy studies were published from 1994 to 2022. The number of citations ranged from 51 to 795, including a total of 14,355 citations as of March 12, 2023. 3 literatures are cited more than 500 times, and 19 pieces of literature are cited more than 200 times (Table 1). When divided into five years, the period with the most significant distribution of literature was 2014–2018, with 35 published articles (Fig. 1). *Annals of Thoracic Surgery* accounted for the highest percentage of articles in the top 100 most cited articles, with 27 articles. According to the latest 2021 Impact Factor (IF) released in 2022, the top 5 journals are *Lancet, Lancet Respiratory Medicine, British Medical Journal, Journal of Clinical Oncology, Journal of Thoracic Oncology* (Table 2). The 100 most cited articles were categorized as 93 original articles (including 82 retrospective research and 11 prospective research) and 7 reviews.

**Table 1:** The top 100 most cited articles on sublobectomy

No	Authors	Article title	Journal	Times cited	Publication year
1	Martini, N	Incidence of Local Recurrence and 2nd Primary Tumors In Resected Stage-I Lung-Cancer	Journal of Thoracic and Cardiovascular Surgery	795	1995
2	Holmes, Ce	Randomized Trial of Lobectomy Versus Limited Resection For T1 N0 Non-Small-Cell Lung-Cancer	Annals of Thoracic Surgery	541	1995
3	Okada, M	Radical Sublobar Resection For Small-Sized Non-Small Cell Lung Cancer: A Multicenter Study	Journal of Thoracic and Cardiovascular Surgery	585	2006
4	Nakamura, K	A Phase III Randomized Trial of Lobectomy Versus Limited Resection For Small-Sized Peripheral Non-Small Cell Lung Cancer (Jcog0802/Wjog4607l)	Japanese Journal of Clinical Oncology	454	2010
5	Grills, Is	Outcomes After Stereotactic Lung Radiotherapy Or Wedge Resection For Stage I Non-Small-Cell Lung Cancer	Journal of Clinical Oncology	345	2010
6	Keenan, Rj	Segmental Resection Spares Pulmonary Function In Patients With Stage I Lung Cancer	Annals of Thoracic Surgery	324	2004
7	Landreneau, Rj	Wedge Resection Versus Lobectomy For Stage I (T1 N0 M0) Non-Small-Cell Lung Cancer	Journal of Thoracic and Cardiovascular Surgery	294	1997
8	El-Sherif, A	Outcomes of Sublobar Resection Versus Lobectomy For Stage I Non-Small Cell Lung Cancer: A 13-Year Analysis	Annals of Thoracic Surgery	300	2006
9	Okada, M	Effect of Tumor Size On Prognosis In Patients With Non-Small Cell Lung Cancer: The Role of Segmentectomy As A Type of Lesser Resection	Journal of Thoracic and Cardiovascular Surgery	292	2005
10	Mery, Cm	Similar Long-Term Survival of Elderly Patients With Non-Small Cell Lung Cancer Treated With Lobectomy Or Wedge Resection Within The Surveillance, Epidemiology, and End Results Database	Chest	272	2005
11	Koike, T	Intentional Limited Pulmonary Resection For Peripheral T1 No Mo Small-Sized Lung Cancer	Journal of Thoracic and Cardiovascular Surgery	263	2003
12	Kodama, K	Intentional Limited Resection For Selected Patients With T1 N0 M0 Non-Small-Cell Lung Cancer: A Single-Institution Study	Journal of Thoracic and Cardiovascular Surgery	255	1997
13	Okada, M	Is Segmentectomy With Lymph Node Assessment An Alternative To Lobectomy For Non-Small Cell Lung Cancer of 2 Cm Or Smaller?	Annals of Thoracic Surgery	253	2001
14	Schuchert, Mj	Anatomic Segmentectomy In The Treatment of Stage I Non-Small Cell Lung Cancer	Annals of Thoracic Surgery	260	2007
15	Altorki, Nk	Sublobar Resection Is Equivalent To Lobectomy For Clinical Stage 1a Lung Cancer In Solid Nodules	Journal of Thoracic and Cardiovascular Surgery	242	2014
16	Suzuki, K	Comparison of Pulmonary Segmentectomy and Lobectomy: Safety Results of A Randomized Trial	Journal of Thoracic and Cardiovascular Surgery	261	2019
17	Warren, Wh	Segmentectomy Versus Lobectomy In Patients With Stage-I Pulmonary-Carcinoma—5-Year Survival and Patterns of Intrathoracic Recurrence	Journal of Thoracic and Cardiovascular Surgery	236	1994
18	Harada, H	Functional Advantage After Radical Segmentectomy Versus Lobectomy For Lung Cancer	Annals of Thoracic Surgery	244	2005
19	Shirvani, Sm	Comparative Effectiveness of 5 Treatment Strategies For Early-Stage Non-Small Cell Lung Cancer In The Elderly	International Journal of Radiation Oncology Biology Physics	205	2012
20	Altorki, Nk	Perioperative Mortality and Morbidity After Sublobar Versus Lobar Resection For Early-Stage Non-Small-Cell Lung Cancer: Post-Hoc Analysis of An International, Randomised, Phase 3 Trial (Calgb/Alliance 140,503)	Lancet Respiratory Medicine	195	2018
21	Zheng, Xp	Survival Outcome After Stereotactic Body Radiation Therapy and Surgery For Stage I Non-Small Cell Lung Cancer: A Meta-Analysis	International Journal of Radiation Oncology Biology Physics	188	2014
22	Shirvani, Sm	Lobectomy, Sublobar Resection, and Stereotactic Ablative Radiotherapy For Early-Stage Non-Small Cell Lung Cancers In The Elderly	Jama Surgery	188	2014
23	Landreneau, Rj	Recurrence and Survival Outcomes After Anatomic Segmentectomy Versus Lobectomy For Clinical Stage I Non-Small-Cell Lung Cancer: A Propensity-Matched Analysis	Journal of Clinical Oncology	197	2014
24	El-Sherif, A	Margin and Local Recurrence After Sublobar Resection of Non-Small Cell Lung Cancer	Annals of Surgical Oncology	189	2007
25	Dai, Cy	Choice of Surgical Procedure For Patients With Non-Small-Cell Lung Cancer < = 1 Cm Or > 1 To 2 Cm Among Lobectomy, Segmentectomy, and Wedge Resection: A Population-Based Study	Journal of Clinical Oncology	180	2016
26	Miller, Dl	Surgical Treatment of Non-Small Cell Lung Cancer 1 Cm Or Less In Diameter	Annals of Thoracic Surgery	172	2002
27	Khullar, Ov	Survival After Sublobar Resection Versus Lobectomy For Clinical Stage Ia Lung Cancer An Analysis From The National Cancer Data Base	Journal of Thoracic Oncology	163	2015
28	Kates, M	Survival Following Lobectomy and Limited Resection For The Treatment of Stage I Non-Small Cell Lung Cancer < = 1 Cm In Size A Review of Seer Data	Chest	160	2011
29	Sienel, W	Sublobar Resections In Stage Ia Non-Small Cell Lung Cancer: Segmentectomies Result In Significantly Better Cancer-Retated Survival Than Wedge Resections	European Journal of Cardio-Thoracic Surgery	152	2008
30	Saji, H	Segmentectomy Versus Lobectomy In Small-Sized Peripheral Non-Small-Cell Lung Cancer (Jcog0802/Wjog4607l): A Multicentre, Open-Label, Phase 3, Randomised, Controlled, Non-Inferiority Trial	Lancet	144	2022
31	Wolf, As	Lobectomy Versus Sublobar Resection For Small (2 Cm Or Less) Non-Small Cell Lung Cancers	Annals of Thoracic Surgery	144	2011
32	Atkins, Bz	Pulmonary Segmentectomy By Thoracotomy Or Thoracoscopy: Reduced Hospital Length of Stay With A Minimally-Invasive Approach	Annals of Thoracic Surgery	151	2007
33	Martin-Ucar, Ae	A Case-Matched Study of Anatomical Segmentectomy Versus Lobectomy For Stage I Lung Cancer In High-Risk Patients	European Journal of Cardio-Thoracic Surgery	150	2005
34	Whitson, Ba	Survival After Lobectomy Versus Segmentectomy For Stage I Non-Small Cell Lung Cancer: A Population-Based Analysis	Annals of Thoracic Surgery	139	2011
35	Nakamura, H	Survival Following Lobectomy Vs Limited Resection For Stage I Lung Cancer: A Meta-Analysis	British Journal of Cancer	130	2005
36	Zemlyak, A	Comparison of Survival After Sub Lobar Resections and Ablative Therapies For Stage I Non-Small Cell Lung Cancer	Journal of The American College of Surgeons	129	2010
37	Shapiro, M	Thoracoscopic Segmentectomy Compares Favorably With Thoracoscopic Lobectomy For Patients With Small Stage I Lung Cancer	Journal of Thoracic and Cardiovascular Surgery	142	2009
38	Cao, C	Could Less Be More?-a Systematic Review and Meta-Analysis of Sublobar Resections Versus Lobectomy For Non-Small Cell Lung Cancer According To Patient Selection	Lung Cancer	127	2015
39	Kilic, A	Anatomic Segmentectomy For Stage I Non-Small Cell Lung Cancer In The Elderly	Annals of Thoracic Surgery	128	2009
40	Zhong, Cx	Comparison of Thoracoscopic Segmentectomy and Thoracoscopic Lobectomy For Small-Sized Stage Ia Lung Cancer	Annals of Thoracic Surgery	135	2012
41	Koike, T	Risk Factor Analysis of Locoregional Recurrence After Sublobar Resection In Patients With Clinical Stage Ia Non-Small Cell Lung Cancer	Journal of Thoracic and Cardiovascular Surgery	116	2013
42	Kodama, K	Oncologic Outcomes of Segmentectomy Versus Lobectomy For Clinical T1a N0 M0 Non-Small Cell Lung Cancer	Annals of Thoracic Surgery	121	2016
43	Sienel, W	Frequency of Local Recurrence Following Segmentectomy of Stage Ia Non-Small Cell Lung Cancer Is Influenced By Segment Locatisation and Width of Resection Margins—Implications For Patient Selection For Segmentectomy	European Journal of Cardio-Thoracic Surgery	119	2007
44	Santos, R	Comparison Between Sublobar Resection and (125)Iodine Brachytherapy After Sublobar Resection In High-Risk Patients With Stage I Non-Small-Cell Lung Cancer	Surgery	109	2003
45	Lee, W	Limited Resection For Non-Small Cell Lung Cancer: Observed Local Control With Implantation of i-125 Brachytherapy Seeds	Annals of Thoracic Surgery	151	2003
46	Fernando, Hc	Lobar and Sublobar Resection With and Without Brachytherapy For Small Stage Ia Non-Small Cell Lung Cancer	Journal of Thoracic and Cardiovascular Surgery	103	2005
47	Schuchert, Mj	Anatomic Segmentectomy For Stage I Non-Small-Cell Lung Cancer: Comparison of Video-Assisted Thoracic Surgery Versus Open Approach	Journal of Thoracic and Cardiovascular Surgery	114	2009
48	Blasberg, Jd	Sublobar Resection A Movement From The Lung Cancer Study Group	Journal of Thoracic Oncology	108	2010
49	Hattori, A	Is Limited Resection Appropriate For Radiologically Solid Tumor In Small Lung Cancers?	Annals of Thoracic Surgery	99	2012
50	Okami, J	Sublobar Resection Provides An Equivalent Survival After Lobectomy In Elderly Patients With Early Lung Cancer	Annals of Thoracic Surgery	102	2010
51	Fan, J	Sublobectomy Versus Lobectomy For Stage I Non-Small-Cell Lung Cancer, A Meta-Analysis of Published Studies	Annals of Surgical Oncology	104	2012
52	Koike, T	Limited Resection For Noninvasive Bronchioloalveolar Carcinoma Diagnosed By Intraoperative Pathologic Examination	Annals of Thoracic Surgery	91	2009
53	Cao, Jl	Survival Rates After Lobectomy, Segmentectomy, and Wedge Resection For Non-Small Cell Lung Cancer	Annals of Thoracic Surgery	98	2018
54	Schuchert, Mj	Anatomic Segmentectomy For The Solitary Pulmonary Nodule and Early-Stage Lung Cancer	Annals of Thoracic Surgery	105	2012
55	Sihoe, Adl	Non-Small Cell Lung Cancer: When To offer Sublobar Resection	Lung Cancer	91	2014
56	Altorki, Nk	Anatomical Segmentectomy and Wedge Resections Are Associated With Comparable Outcomes For Patients With Small CT1N0 Non-Small Cell Lung Cancer	Journal of Thoracic Oncology	86	2016
57	Mohiuddin, K	Relationship Between Margin Distance and Local Recurrence Among Patients Undergoing Wedge Resection For Small (< = 2 Cm) Non-Small Cell Lung Cancer	Journal of Thoracic and Cardiovascular Surgery	95	2014
58	Smith, Cb	Survival After Segmentectomy and Wedge Resection In Stage I Non-Small-Cell Lung Cancer	Journal of Thoracic Oncology	96	2013
59	Veluswamy, Rr	Limited Resection Versus Lobectomy For Older Patients With Early-Stage Lung Cancer: Impact of Histology	Journal of Clinical Oncology	94	2015
60	Fernando, Hc	Impact of Brachytherapy On Local Recurrence Rates After Sublobar Resection: Results From Acosog z4032 (Alliance), A Phase III Randomized Trial For High-Risk Operable Non-Small-Cell Lung Cancer	Journal of Clinical Oncology	78	2014
61	Matsuo, Y	Comparison of Long-Term Survival Outcomes Between Stereotactic Body Radiotherapy and Sublobar Resection For Stage I Non-Small-Cell Lung Cancer In Patients At High Risk For Lobectomy: A Propensity Score Matching Analysis	European Journal of Cancer	77	2014
62	Kent, M	Segmentectomy Versus Wedge Resection For Non-Small Cell Lung Cancer In High-Risk Operable Patients	Annals of Thoracic Surgery	78	2013
63	Nakamura, H	Comparison of The Surgical Outcomes of Thoracoscopic Lobectomy, Segmentectomy, and Wedge Resection For Clinical Stage I Non-Small Cell Lung Cancer	Thoracic and Cardiovascular Surgeon	83	2011
64	Ohta, Y	Can Tumor Size Be A Guide For Limited Surgical Intervention In Patients With Peripheral Non-Small Cell Lung Cancer? Assessment From The Point of View of Nodal Micrometastasis	Journal of Thoracic and Cardiovascular Surgery	80	2001
65	Yendamuri, S	Temporal Trends In Outcomes Following Sublobar and Lobar Resections For Small (< = 2 Cm) Non-Small Cell Lung Cancers-a Surveillance Epidemiology End Results Database Analysis	Journal of Surgical Research	81	2013
66	Carr, Sr	Impact of Tumor Size On Outcomes After Anatomic Lung Resection For Stage 1a Non-Small Cell Lung Cancer Based On The Current Staging System	Journal of Thoracic and Cardiovascular Surgery	78	2012
67	Nakata, M	Objective Radiologic Analysis of Ground-Glass Opacity Aimed At Curative Limited Resection For Small Peripheral Non-Small Cell Lung Cancer	Journal of Thoracic and Cardiovascular Surgery	72	2005
68	Paul, S	Long Term Survival With Stereotactic Ablative Radiotherapy (Sabr) Versus Thoracoscopic Sublobar Lung Resection In Elderly People: National Population Based Study With Propensity Matched Comparative Analysis	Bmj-British Medical Journal	69	2016
69	Cao, C	Meta-Analysis of Intentional Sublobar Resections Versus Lobectomy For Early Stage Non-Small Cell Lung Cancer	Annals of Cardiothoracic Surgery	75	2014
70	Yamashita, S	Thoracoscopic Segmentectomy For T1 Classification of Non-Small Cell Lung Cancer: A Single Center Experience	European Journal of Cardio-Thoracic Surgery	81	2012
71	Zhang, Y	Meta-Analysis of Lobectomy, Segmentectomy, and Wedge Resection For Stage I Non-Small Cell Lung Cancer	Journal of Surgical Oncology	76	2015
72	Subramanian, M	Long-Term Results For Clinical Stage Ia Lung Cancer: Comparing Lobectomy and Sublobar Resection	Annals of Thoracic Surgery	68	2018
73	Bedetti, B	Segmentectomy Versus Lobectomy For Stage I Non-Small Cell Lung Cancer: A Systematic Review and Meta-Analysis	Journal of Thoracic Disease	76	2017
74	Fernando, Hc	American College of Surgeons Oncology Group z4099/Radiation Therapy Oncology Group 1021: A Randomized Study of Sublobar Resection Compared With Stereotactic Body Radiotherapy For High-Risk Stage I Non-Small Cell Lung Cancer	Journal of Thoracic and Cardiovascular Surgery	64	2012
75	Speicher, Pj	Sublobar Resection For Clinical Stage Ia Non-Small-Cell Lung Cancer In The United States	Clinical Lung Cancer	62	2016
76	Nomori, H	Segmentectomy For Selected CT1N0M0 Non-Small Cell Lung Cancer: A Prospective Study At A Single Institute	Journal of Thoracic and Cardiovascular Surgery	73	2012
77	Shiraishi, T	Video-Assisted Thoracoscopic Surgery (Vats) Segmentectomy For Small Peripheral Lung Cancer Tumors—Intermediate Results	Surgical Endoscopy and Other Interventional Techniques	68	2004
78	Razi, Ss	Sublobar Resection Is Equivalent To Lobectomy For T1a Non-Small Cell Lung Cancer In The Elderly: A Surveillance, Epidemiology, and End Results Database Analysis	Journal of Surgical Research	65	2016
79	Hwang, Y	Comparison of Thoracoscopic Segmentectomy and Thoracoscopic Lobectomy On The Patients With Non-Small Cell Lung Cancer: A Propensity Score Matching Study	European Journal of Cardio-Thoracic Surgery	75	2015
80	Ghaly, G	Video-Assisted Thoracoscopic Surgery Is A Safe and Effective Alternative To Thoracotomy For Anatomical Segmentectomy In Patients With Clinical Stage I Non-Small Cell Lung Cancer	Annals of Thoracic Surgery	70	2016
81	Watanabe, A	Feasibility of Video-Assisted Thoracoscopic Surgery Segmentectomy For Selected Peripheral Lung Carcinomas	European Journal of Cardio-Thoracic Surgery	69	2009
82	Handa, Y	Surgical Outcomes of Complex Versus Simple Segmentectomy For Stage I Non-Small Cell Lung Cancer	Annals of Thoracic Surgery	59	2019
83	Yendamuri, S	Is Sublobar Resection Sufficient For Carcinoid Tumors?	Annals of Thoracic Surgery	55	2011
84	Fernando, Hc	Thirty- and Ninety-Day Outcomes After Sublobar Resection With and Without Brachytherapy For Non-Small Cell Lung Cancer: Results From A Multicenter Phase III Study	Journal of Thoracic and Cardiovascular Surgery	53	2011
85	Nomori, H	Sentinel Node Navigation Segmentectomy For Clinical Stage Ia Non-Small Cell Lung Cancer	Journal of Thoracic and Cardiovascular Surgery	52	2007
86	Aokage, K	Limited Resection For Early-Stage Non-Small Cell Lung Cancer As Function-Preserving Radical Surgery: A Review	Japanese Journal of Clinical Oncology	61	2017
87	Fiorelli, A	Sublobar Resection Versus Lobectomy For Stage I Non-Small Cell Lung Cancer: An Appropriate Choice In Elderly Patients?	Surgery Today	52	2016
88	Birdas, Tj	Sublobar Resection With Brachytherapy Versus Lobectomy For Stage Ib Nonsmall Cell Lung Cancer	Annals of Thoracic Surgery	56	2006
89	Watanabe, T	Intentional Limited Resection For Small Peripheral Lung Cancer Based On Intraoperative Pathologic Exploration	General Thoracic and Cardiovascular Surgery	50	2005
90	Konaka, C	Peripheral Non-Small Cell Lung Cancers 2.0 Cm Or Less In Diameter: Proposed Criteria For Limited Pulmonary Resection Based Upon Clinicopathological Presentation	Lung Cancer	49	1998
91	Deng, Hy	Radiotherapy, Lobectomy Or Sublobar Resection? A Meta-Analysis of The Choices For Treating Stage I Non-Small-Cell Lung Cancer	European Journal of Cardio-Thoracic Surgery	50	2017
92	Smith, Cb	Comparative Outcomes of Elderly Stage I Lung Cancer Patients Treated With Segmentectomy Via Video-Assisted Thoracoscopic Surgery Versus Open Resection	Journal of Thoracic Oncology	50	2014
93	Nishio, W	Re-Assessment of Intentional Extended Segmentectomy For Clinical T1aN0 Non-Small Cell Lung Cancer	Annals of Thoracic Surgery	52	2016
94	Yendamuri, S	Effect of The Number of Lymph Nodes Examined On The Survival of Patients With Stage I Non-Small Cell Lung Cancer Who Undergo Sublobar Resection	Journal of Thoracic and Cardiovascular Surgery	49	2018
95	Dziedzic, R	Stage I Non-Small-Cell Lung Cancer: Long-Term Results of Lobectomy Versus Sublobar Resection From The Polish National Lung Cancer Registry	European Journal of Cardio-Thoracic Surgery	54	2017
96	De Zoysa, Mk	Is Limited Pulmonary Resection Equivalent To Lobectomy For Surgical Management of Stage I Non-Small-Cell Lung Cancer?	Interactive Cardiovascular and Thoracic Surgery	47	2012
97	Okumura, M	Factors Associated With Outcome of Segmentectomy For Non-Small Cell Lung Cancer: Long-Term Follow-Up Study At A Single Institution In Japan	Lung Cancer	66	2007
98	Koike, T	Lobectomy Versus Segmentectomy In Radiologically Pure Solid Small-Sized Non-Small Cell Lung Cancer	Annals of Thoracic Surgery	58	2016
99	Kwan, Sw	Thermal Ablation Matches Sub Lobar Resection Outcomes In Older Patients With Early-Stage Non-Small Cell Lung Cancer	Journal of Vascular and Interventional Radiology	47	2014
100	Chan, Eg	Preoperative (3-Dimensional) Computed Tomography Lung Reconstruction Before Anatomic Segmentectomy Or Lobectomy For Stage I Non-Small Cell Lung Cancer	Journal of Thoracic and Cardiovascular Surgery	51	2015

**Table 2:** Journals and their impact factors included in the 100 most cited articles on sublobectomy

Rank	Journal	Articles	Impact factor
1	Annals Of Thoracic Surgery	27	5.102
2	Journal Of Thoracic And Cardiovascular Surgery	23	6.439
3	European Journal Of Cardio-Thoracic Surgery	8	4.534
4	Journal Of Clinical Oncology	5	50.717
5	Journal Of Thoracic Oncology	5	20.121
6	Lung Cancer	4	6.081
7	Chest	2	10.262
8	International Journal Of Radiation Oncology Biology Physics	2	8.013
9	Annals Of Surgical Oncology	2	4.339
10	Japanese Journal Of Clinical Oncology	2	2.925
11	Journal Of Surgical Research	2	2.417
12	Lancet	1	202.731
13	Lancet Respiratory Medicine	1	102.642
14	British Medical Journal	1	93.333
15	Jama Surgery	1	16.681
16	European Journal Of Cancer	1	10.002
17	British Journal Of Cancer	1	9.075
18	Journal Of The American College Of Surgeons	1	6.5321
19	Clinical Lung Cancer	1	4.84
20	Annals Of Cardiothoracic Surgery	1	4.617
21	Surgery	1	4.348
22	Journal Of Vascular And Interventional Radiology	1	3.682
23	Surgical Endoscopy And Other Interventional Techniques	1	3.453
24	Journal Of Thoracic Disease	1	3.005
25	Journal Of Surgical Oncology	1	2.885
26	Surgery Today	1	2.54
27	Interactive Cardiovascular And Thoracic Surgery	1	1.978
28	Thoracic And Cardiovascular Surgeon	1	1.756
29	General Thoracic And Cardiovascular Surgery	1	1.227

**Fig. 2:**
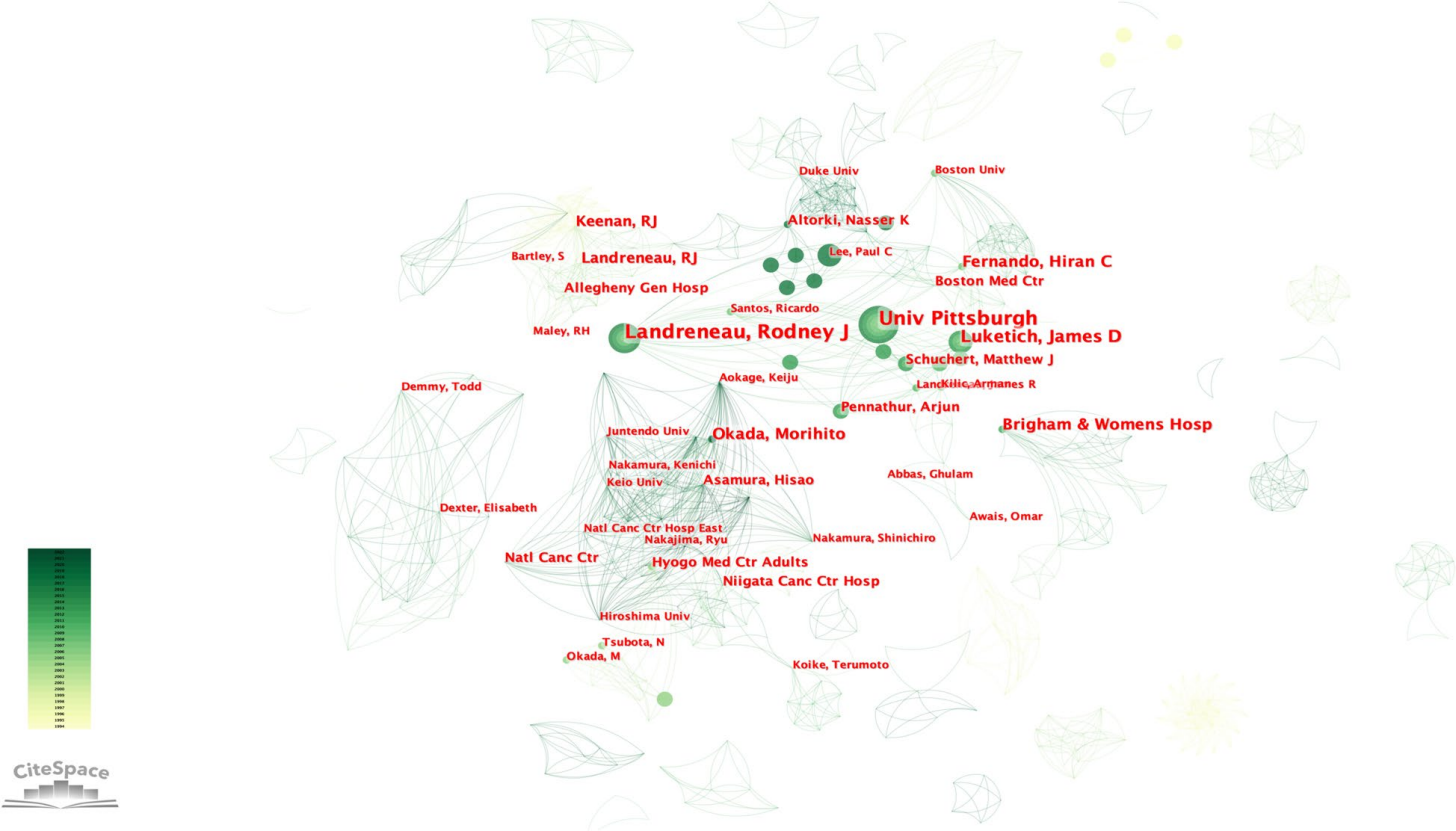
Citespace network of co-cited authorship and organization in the field of sublobectomy for NSCLC. Every circle represents one author or organization. Size of circle is positively linked to cited counts of the authors and organizations, links between two circles represents a collaboration between two authors or organizations on the same article. Frequency of collaborations were presented by line thickness

**Fig. 3:**
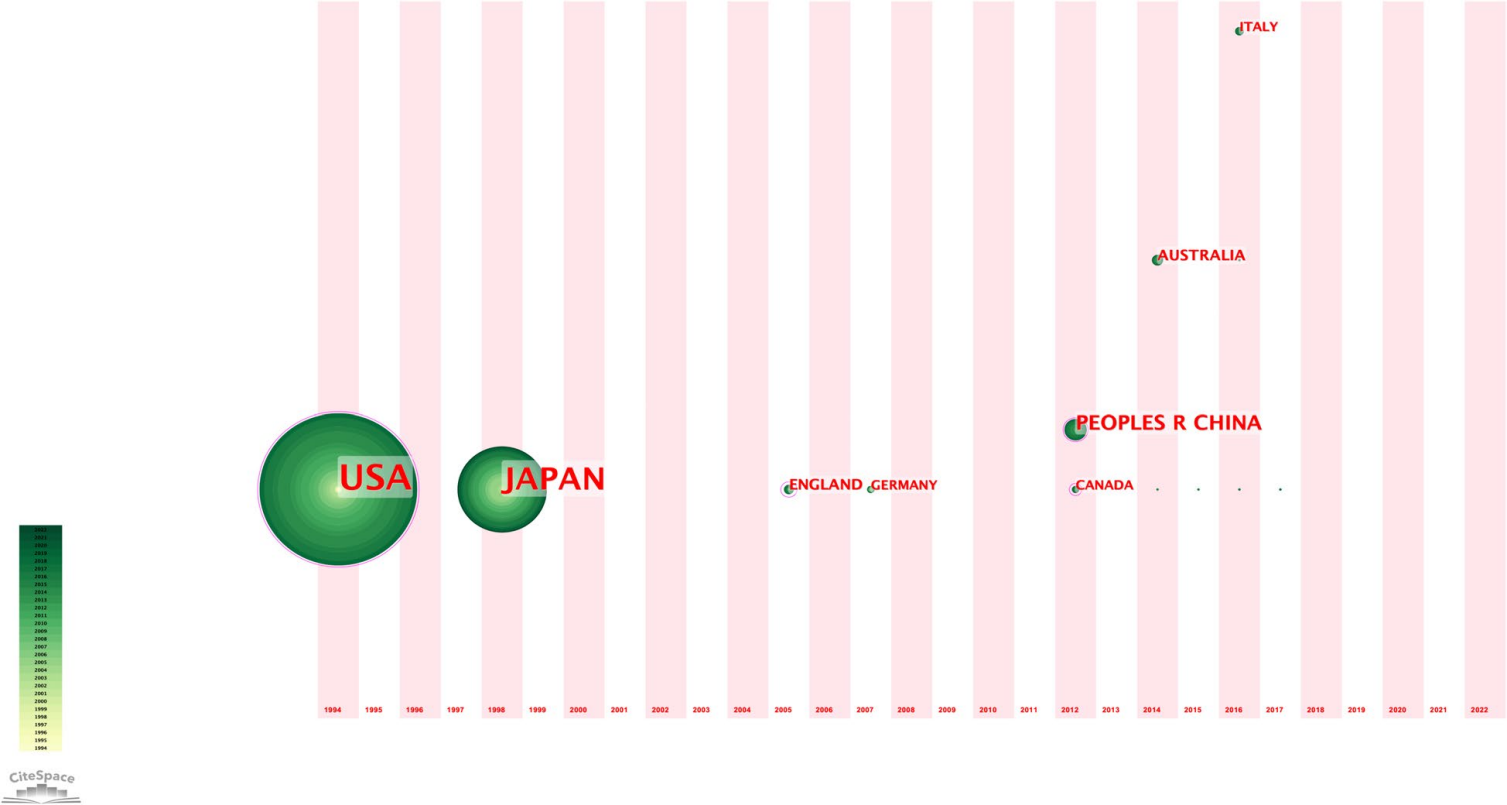
Time-Zone view of high-frequency countries of top 100 most cited articles. The image displays the evolution of high-frequency countries of top 100 most cited articles over time, from 1994 to 2022. The size of the nodes corresponds to the occurrence frequency of these keywords

Among authors of the top 100 most cited works, the top five are Okada Morihito, Landreneau Rodney J, Luketich James D, Fernando Hiran C, Keenan Rj and Landreneau Rj (tied for 5th), with 1410, 1393, 1264, 952, and 829 citations, respectively. Considering both the number of articles included, the top five authors with the most published articles are Landreneau Rodney J, Luketich James D, Fernando Hiran C, Schuchert Matthew J, Okada Morihito and Pennathur Arjun (tied for 5th), with 11, 9,7,7and 6 articles (Table 3). The 100 most cited articles come from 155 organizations, with the top five being University of Pittsburgh, Brigham & Women’s Hospital, National Cancer Center, Mayo Clinic and Niigata Cancer Center (Fig. 2). The 100 most cited articles come from 13 countries. The top five countries of most cited papers are the U.S.A, Japan, China, Australia, and England (Table 4 & Fig. 3).

**Table 3:** Authors that contributed 4 or more articles in 100 most cited articles on sublobectomy

Rank	Author	Articles	Citations
1	Landreneau Rodney J	11	1393
2	Luketich James D	9	1264
3	Fernando Hiran C	7	952
4	Schuchert Matthew J	7	809
5	Okada Morihito	6	1410
6	Pennathur Arjun	6	765
7	Keenan Rj	5	829
8	Landreneau Rj	5	829
9	Wisnivesky Juan P	5	469
10	Asamura Hisao	4	822
11	Saji Hisashi	4	822
12	Tsuboi Masahiro	4	822
13	Altorki Nasser K	4	547
14	Suzuki Kenji	4	504
15	Landreneau James R	4	483
16	Swanson Scott J	4	410
17	Koike Terumoto	4	286

**Table 4:** Countries of origin with 3 or more papers included in the 100 most cited articles on sublobectomy

Rank	Country	Articles	Citations
1	U.S.A	53	7762
2	Japan	31	4026
3	China	9	817
4	Australia	4	537
5	England	4	410
6	Germany	3	304
7	Canada	3	315
8	Italy	3	280

**Fig. 4:**
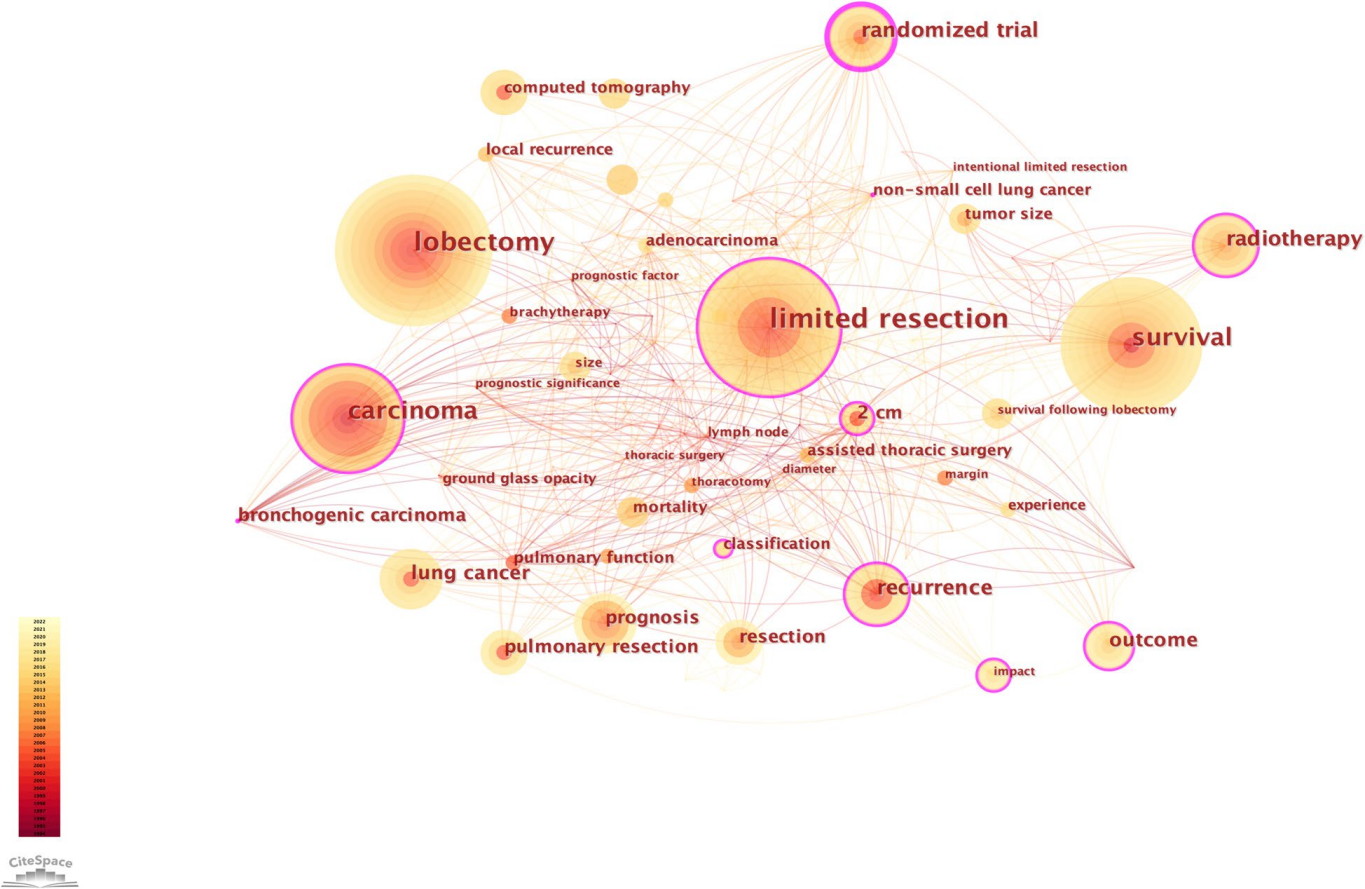
Citespace network of co-occurrence networks of keywords in the field of sublobectomy for NSCLC. Every circle represents one keyword. Size of circle is positively linked to cited counts of the keywords, links between two circles represents a collaboration between two keywords on the same article. Frequency of collaborations were presented by line thickness

Of the 100 articles in this study, the top 10 keywords are limited resection, lobectomy, survival, carcinoma, recurrence, randomized trial, radiotherapy, lung cancer, outcome, 2 cm. Through CiteSpace analysis, we found that limited resection, lobectomy, carcinoma, randomized trial and survival became high-density keywords (Fig. 4).

## Discussion

With the gradual popularization of chest computed tomography (CT) as a means of health checkups, more and more NSCLC are being discovered in the form of small nodules. When facing this situation, both young and elderly patients need to completely remove the tumor while preserving as much lung function as possible. Therefore, sublobectomy has gradually entered the field of vision of thoracic surgeons. Recently, more and more studies have focused on sublobectomy for the treatment of NSCLC. We have constructed this bibliometric analysis specifically for sublobectomy, trying to sort out the current status of existing sublobectomy research by summarizing the most 100 cited articles on sublobectomy and providing a practical reference for future sublobectomy research. While there have been recent bibliometric studies in related fields [13], our research offers a fresh and valuable contribution to the literature. Our study's significance stems from its comprehensive coverage of a vast array of publications and its up-to-date analysis, which reflects the latest trends in the field.

Several studies have demonstrated that sublobectomy can achieve similar oncological outcomes as lobectomy for NSCLC while preserving more lung function [3, 11]. A recent meta-analysis by Zhang et al. [14] showed that segmentectomy had a lower incidence of postoperative complications and better preservation of pulmonary function compared to lobectomy. However, some studies have reported a higher local recurrence rate after sublobectomy [7, 15]. Therefore, the choice between sublobectomy and lobectomy should be based on tumor size, location, and patient's lung function.

The 2 cm size criterion for sublobectomy has been widely accepted in clinical practice. This cutoff is based on the assumption that tumors smaller than 2 cm have a lower risk of lymph node metastasis and can be adequately treated with sublobectomy [16]. However, recent studies have challenged this notion, suggesting that sublobectomy may be appropriate for tumors larger than 2 cm in selected patients [6, 17]. For example, a study by Altorki et al. [18] demonstrated that segmentectomy was non-inferior to lobectomy for tumors up to 3 cm in size; The JCOG1211 study found that ground-glass nodules below 3 cm could also be considered for treatment with sublobectomy [9]. Further research is needed to refine the selection criteria for sublobectomy and to identify the optimal surgical approach for individual patients.

The importance of prospective randomized controlled trials (RCTs) in the field of sublobectomy cannot be overstated. RCTs provide the highest level of evidence for clinical decision-making and can help to establish the optimal surgical approach for early-stage NSCLC [19]. Several RCTs, such as the JCOG0802/WJOG4607L study [8], JCOG1211 study [9] and the CALGB 140503 study [10], have demonstrated that sublobectomy is not inferior to lobectomy for small-sized non-small cell lung cancer. Given the clinical significance of the above study, the findings are likely to rewrite future guidelines for the surgical treatment of NSCLC.

Sublobectomy and radiotherapy are two common treatment options for early-stage NSCLC. Radiotherapy uses high-energy radiation to destroy cancer cells, with stereotactic body radiotherapy (SBRT) being a popular choice for inoperable patients [20]. However, segmentectomy may offer better regional control comparing SBRT [21]. Radiotherapy has the advantage of being a non-invasive treatment, making it more suitable for patients with poor lung function or other comorbidities [22]. The choice between sublobectomy and radiotherapy should be based on individual patient factors, including tumor size, location, and overall health status.

The United States and Japan are the top two countries with the most highly cited publications. Both countries have made significant contributions to the development and popularization of segmentectomy. Japanese researchers have been pioneers in the field, with several landmark studies on sublobectomy published by Japanese institutions [23, 24], and recently Japanese researchers have also released blockbuster research results [8, 9]. In the United States, the National Cancer Institute has sponsored several clinical trials on segmentectomy, such as the ACOSOG Z4032 trial [25] and the aforementioned CALGB 140503 study [10]. The collaboration between researchers from these two countries has greatly advanced our understanding of segmentectomy and its role in the treatment of early-stage NSCLC. It is noteworthy that the largest number of studies on sublobectomy were published between 2014–2018. We believe this is related to the exploration of optimal surgical approaches for early stage NSCLC that began in the early 21st century, as well as the widespread adoption of minimally invasive thoracic surgery techniques.

In the realm of bibliometric analysis, it is imperative to acknowledge the potential limitations and drawbacks that may arise in the course of conducting research. While the quantitative assessment of scientific literature offers valuable insights into the development and dissemination of knowledge, it is not without its shortcomings. One of the primary concerns in this domain is the overemphasis on bibliometric indicators, such as citation counts and impact factors, which may inadvertently lead to a skewed representation of research quality and significance. Furthermore, the inherent biases in citation practices, including self-citation and preferential attachment, can exacerbate the disparities in the visibility and recognition of scholarly works. Additionally, the reliance on quantitative metrics may overlook the nuances and complexities of scientific research, as it fails to capture the qualitative aspects of knowledge production, such as the context, novelty, and interdisciplinary nature of the studies.

## Conclusion

The first 100 most cited articles in the field of sublobectomy research were included in the bibliometric analysis, and a series of analyses were conducted. Most of the top 100 most cited articles are original and dominated by retrospective research. Of the included literature, *Annals of Thoracic Surgery* was the journal with the most publications The most published and cited works are from the United States. In recent years, research on sublobectomy for NSCLC has gradually shifted focus to patient prognosis and comparison of efficacy with other treatment modalities. Researchers have attempted to improve the evidence-based medicine level of sublobectomy through prospective clinical trials, in order to establish its role in the treatment of NSCLC. As the inaugural bibliometric analysis in the field of sublobectomy, our study not only pioneers the way for future research but also surfaces novel focal points that may captivate the attention of subsequent investigators. We anticipate that our findings will stimulate a deeper exploration into these areas, ultimately leading to the discovery of additional clinically meaningful outcomes within the domain of sublobectomy.

## Data Availability

Data can be provided upon request.

## Abbreviations

NSCLC: Non-small cell lung cancer
WoS: Web of Science
IF: Impact Factor
RCTs: Randomized controlled trials
SBRT: Stereotactic body radiotherapy
